# Leaves of *Moringa oleifera* Are Potential Source of Bioactive Compound β-Carotene: Evidence from In Silico and Quantitative Gene Expression Analysis

**DOI:** 10.3390/molecules28041578

**Published:** 2023-02-07

**Authors:** Ghazala Muteeb, Mohammad Aatif, Mohd Farhan, Abdulrahman Alsultan, Adil Alshoaibi, Mir Waqas Alam

**Affiliations:** 1 Department of Nursing, College of Applied Medical Science, King Faisal University, Al-Ahsa 31982, Saudi Arabia; 2 Department of Public Health, College of Applied Medical Sciences, King Faisal University, Al-Ahsa 31982, Saudi Arabia; 3 Department of Basic Sciences, Preparatory Year Deanship, King Faisal University, Al-Ahsa 31982, Saudi Arabia; 4 Department of Biomedical Sciences, College of Medicine, King Faisal University, Al-Ahsa 31982, Saudi Arabia; 5 Department of Physics, College of Science, King Faisal University, Al-Ahsa 31982, Saudi Arabia

**Keywords:** β-carotene genes, bioactive compounds, gene expression, genomics, transcriptomics, moringa oleifera, real time PCR, RNA and cDNA analysis

## Abstract

*Moringa oleifera* is rich in bioactive compounds such as beta-carotene, which have high nutritional values and antimicrobial applications. Several studies have confirmed that bioactive-compound-based herbal medicines extracted from the leaves, seeds, fruits and shoots of *M. oleifera* are vital to cure many diseases and infections, and for the healing of wounds. The β-carotene is a naturally occurring bioactive compound encoded by zeta-carotene desaturase (ZDS) and phytoene synthase (PSY) genes. In the current study, computational analyses were performed to identify and characterize ZDS and PSY genes retrieved from *Arabidopsis thaliana* (as reference) and these were compared with the corresponding genes in *M. oleifera, Brassica napus*, *Brassica rapa*, *Brassica oleracea* and *Bixa orellana*. The BLAST results revealed that all the plant species considered in this study encode β-carotene genes with 80–100% similarity. The Pfam analysis on β-carotene genes of all the investigated plants confirmed that they belong to the same protein family and domain. Similarly, phylogenetic analysis revealed that β-carotene genes of *M. oleifera* belong to the same ancestral class. Using the ZDS and PSY genes of *Arabidopsis thaliana* as a reference, we conducted qRT-PCR analysis on RNA extracted from the leaves of *M. oleifera*, *Brassica napus*, *Brassica rapa* and *Bixa orellana*. It was noted that the most significant gene expression occurred in the leaves of the studied medicinal plants. We concluded that not only are the leaves of *M. oleifera* an effective source of bioactive compounds including beta carotene, but also the leaves of *Brassica napus*, *Brassica rapa* and *Bixa orellana* can be employed as antibiotics and antioxidants against bacterial or microbial infections.

## 1. Introduction

Plant products play very important roles in public health. According to estimates, about 10% of the 258,650 higher plant species have been found to have potential for the treatment of ailments in humans and animals [1]. In most developing countries, traditional medicines and medicinal plants have been widely used on a normative basis for the maintenance of good health [2]. Moreover, industrialized societies have shown increasing reliance on medicinal plant extracts for the development of various traditional chemotherapeutics, drugs and herbal remedies [3]. Meanwhile, on account of the increasing costs for the maintenance of personal health in low-income countries, herbal medications have become more prevalent for the treatment of various diseases [3].

Most of the medicinal plants, such as *Moringa oleifera*, *Cassia fistula* Linn, *Lawsonia inermis*, *Azadirachta indica* and *Ricinus communis*, contain a large number of bioactive compounds in their leaves [4]. *Moringa oleifera* is one of the well-known and broadly distributed species of a mono-generic family [5]. The leaves of *Moringa* produce thiocarbamate glycosides, mustard oil glycosides and nitriles, bioactive compounds which have been found to be responsible for lowering blood pressure. Meanwhile, the leaves and roots have been extensively used pharmacologically as its ethanol extract constituents show antispasmodic activities via calcium channel blockade [6]. In addition, the extracts of the leaves balance thyroid hormones, which is useful for the treatment of hyperthyroidism, and they also show antioxidant activities [7]. 

Carotenoids are organic pigments found in a plant’s chloroplast and photosynthetic organisms like cyanobacteria, fungi and algae. They are chemical structures having multiple carbon chains classified as: (i) oxygen derivative xanthophylls like zeaxanthin and lutein; and (ii) carotenes like lycopene, α-carotenes and β-carotenes, which consist of hydrogen and carbon. Beta carotene is the most significant member of the carotenoid family and its strongly red–orange-colored pigment is abundantly found in fruity plants [8]. It is regarded as a major element of human diet and the main source of vitamin A [9]. 

Comprehensive research into gene expression analysis for bioactive compounds has provided us with remarkable knowledge, which has been beneficial for predicting functional genes. The bioactive components of food can interact in multiple ways with genes and can alter the expression of phenotypes [10]. Undeniably, bioactive genes can influence metabolism, absorption or assimilation, also known as nutritional transcriptomics, as well as site of action, and thus affect overall body response [11]. The *M. oleifera* leaves contain different types of bioactive compounds such as β-carotene, polyphenols, flavonoids, carotenoids, alkaloids, saponins, phenolic acid, vitamins and phytates, and they are rich sources of polyphenols and flavonoids [12]. 

Moreover, the epigenome comprising different mechanisms, e.g., DNA methylation, remodeling, histone tail modifications, chromatin microRNAs and long non-coding RNAs, interacts with environmental factors such as nutrition, pathogens and climate to influence the expression profiles of genes and the emergence of specific phenotypes [13]. Multi-level interactions between the genome, epigenome and environmental factors might occur. Furthermore, numerous lines of evidence suggest the influence of epigenome variation on health and production. The expression of eukaryotic genes is temporarily and multidimensionally controlled [14]. Only a relatively small set of the entire genome is expressed in each type of tissue, and the expression of genes depends on the stage of development. Therefore, gene expression is specific to each tissue [15]. In addition, the number of gene products that are made in the same tissue, as well as in other tissues that make that product, regulates the expression of that gene. One of the basic activities is the study of genes and proteins related to economic traits and their study at the cellular or chromosomal level [16].

The aim of the current research was to characterize and identify plant-based bioactive compound gene expression in *M. oleifera* with advanced bioinformatics tools and qRT-PCR technique, so that these compounds can be used as antibiotic and antisepsis drugs to improve medication and clinical results.

## 2. Results

### 2.1. Retrieval of Zeta-Carotene Desaturase (ZDS) and Phytoene Synthase (PSY) Genes

Using the genome of *Arabidopsis thaliana* as reference, the ZDS and PSY genes were retrieved from the TAIR database. The genes were searched using the gene class symbol database at TAIR. The TAIR data showed that the ZDS gene was submitted on 19 June 2003 with locus tag AT3G04870 and reviewed by David Meinke from Oklahoma State University. Similarly, TAIR data showed that phytoene synthase, abbreviated as PSY, was submitted with locus tag AT1G06570 and reviewed by David Meinke from Oklahoma State University. Using BLAST at the NCBI, the sequence as well as the identity of the ZDS and PSY genes were confirmed. 

### 2.2. Sequencing of ZDS and PSY Genes of M. oleifera and Submission to NCBI Database

Around 3 Kb of PCR product of the ZDS and PSY genes were sequenced using Illumina MiSeq (Macrogen, Korea). The PCR primer binding sites were excluded by editing and manually corrected using MEGA 7 version software. Full sequences of the *M. oleifera* ZDS and PSY genes were deposited at the NCBI to be registered in GenBank and they received the accession nos. OQ161193 and OQ161194, respectively.

### 2.3. Multiple Sequence Alignments and Phylogenetic Analysis

Multiple sequence alignment refers to the process of aligning the sequences of biologically applicable length using computational algorithms. The gene sequences for the ZDS and PSY of *M. oleifera* were aligned locally by the NCBI with *A. thalina, B. napus, B. rapa* and *B. oleracea*. The results showed that the ZDS of *M. oleifera* has similarity with *A. thalina* (98.0%), *B. napus* (83.4%), *B. rapa* (76.6%) and *B. oleracea* (86.0%). Similarly, the results showed that the PSY of *M. oleifera* has similarity with *A. thalina* (97.5%), *B. napus* (83.5%), *B. rapa* (82.3%) and *B. oleracea* (83.5%). Overall results showed that the bioactive gene sequences of the ZDS and PSY of *M. oleifera*, on the basis of a higher matching index with the bioactive genes of the four plants species *A. thalina*, *Brassica napus*, *Brassica rapa* and *Brassica oleracea*, exhibited 76–100% identity or similarity (Table 1).

**Figure 1:**
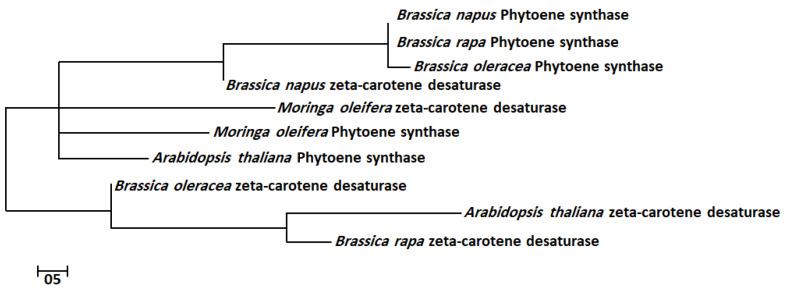
Molecular phylogenetic analysis of ZDS and PSY bioactive genes by maximum likelihood method. The evolutionary history of ZDS and PSY bioactive genes was inferred by using the maximum likelihood method with 1000 bootstraps based on the Tamura–Nei model.

The bioactive gene sequences of ZDS and PSY were aligned by MEGA7-calculated best matches of selected sequences, which showed high similarity among aligned nucleotides sequences. The genes PSY and ZDS had a high tendency to show nucleotide sequences closely similar to *A. thaliana*. The evolutionary history of the ZDS and PSY bioactive genes was inferred by using the maximum likelihood method with 1000 bootstraps based on the Tamura–Nei model in a traditional branch style. These trees show a common ancestral order among all bioactive genes of the query plants (Figure 1). The results of these evolutionary trees showed that the ZDS and PSY of *M. oleifera* are in the same cluster with *A. thaliana*, followed by *Brassica napus*, *Brassica rapa* and *Brassica oleracea*, indicating the close association of these plant families and their importance as medicinal plants. 

**Figure 2:**
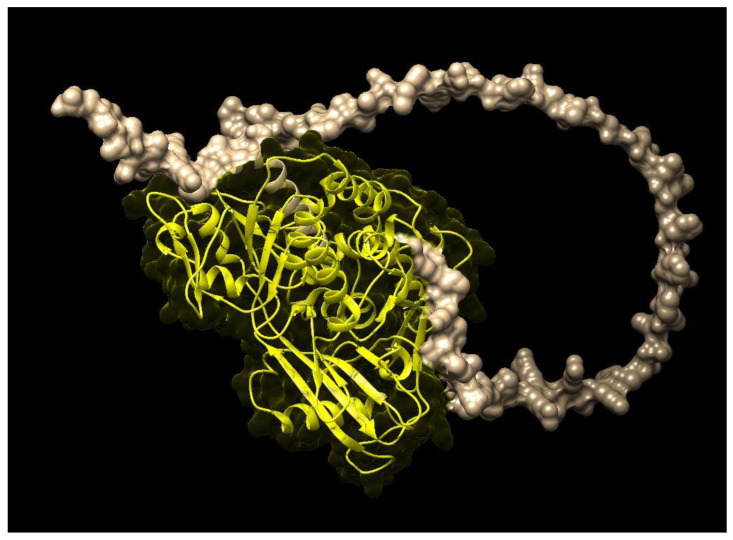
Three-dimensional structure of *M. oleifera* zeta-carotene desaturase (ZDS).

The Uniport, EMBL and Pfam databases were used to retrieve the domain as well as the protein families of these genes. The results showed that the protein families of the ZDS and PSY of *M. oleifera* were similar to those of *A. thaliana, Brassica napus, Brassica rapa* and *Brassica oleracea*. Moreover, analysis using the Pfam and CDD databases exhibited that the same flavin-containing amine oxidoreductase PLN02487 and PLN02632 domains having protein–protein interactions in both ZDS and PSY genes control different physiological functions. The three-dimensional structure of the ZDS (Figure 2) of *M. oleifera*, like *A. thaliana*, carries two consecutive desaturations (introduction of double bonds) at positions C-7 and C-7 double bond, which shows stereoselectivity toward trans C15-C15′ zeta-carotene double bond. ZDS plays a crucial role in plant growth and development and is essential for the biosynthesis of carotenoids. Along with several other biological and molecular functions, it catalyzes the conversion of zeta-carotene to lycopene via the intermediary of neurosporene.

**Figure 3:**
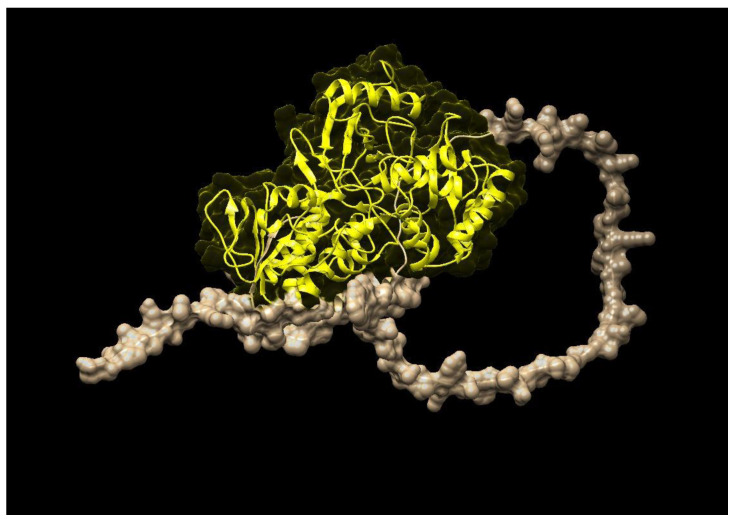
Three-dimensional structure of *M. oleifera* phytoene synthase (PSY).

**Figure 4:**
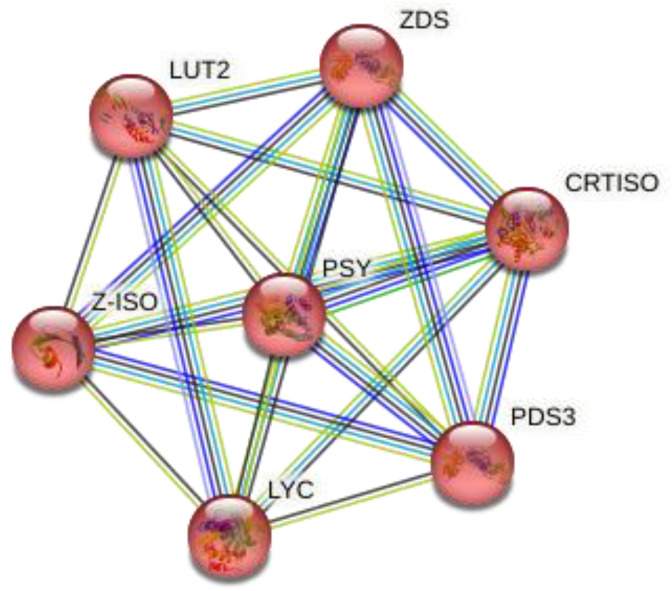
Protein–protein network interaction of *Moringa oleifera*, *Arabidopsis thaliana*, *Brassica napus*, *Brassica rapa* and *Brassica oleracea*.

Similarly, Figure 3 represents the three-dimensional structure of PSY (phytoene synthase), which converts phytoene into zeta-carotene via the intermediary of phytofluene by the symmetrical introduction of two double bonds at the C-11 and C-11′ positions of phytoene. The zeta-carotene has a central C15-C15′ double bond in the cis configuration and it requires isomerization before being recognized as substrate by ZDS, the next enzyme in the lycopene biosynthesis pathway. The STRING interaction analysis (Figure 4) further explained that both ZDS and PSY have close association in various metabolic pathways as well as molecular function, which leads to production of beta-carotene and zeta-carotene bioactive compounds. For instance, lycopene epsilon cyclase (LUT2) catalyzes the single epsilon-cyclization reaction which converts lycopene to delta-carotene. Similarly, Z-ISO (15-cis-zeta-carotene isomerase) is involved in the biosynthesis of carotenoids, and CRTISO is involved in carotenoid and chloroplast regulation.

Following the confirmation of the similarity with most of the medicinal plant’s genes, the ZDS and PSY of *A. thaliana* were used in qRT-PCR analysis of the *M. oleifera* plant’s leaves. 

### 2.4. RNA Extraction and cDNA Preparation

**Figure 5:**
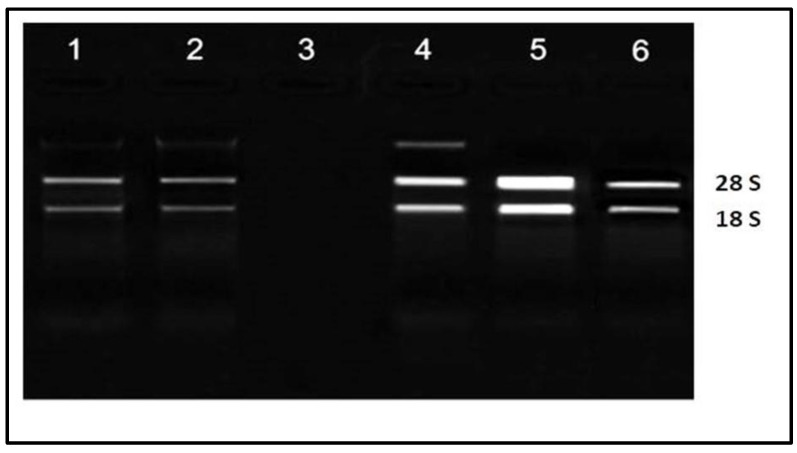
The 28S and 18S RNA bands of extracted RNA from leaves of *Moringa oleifera*.

The integrity of the RNA extracted from the leaves of *M. oleifera* was checked by loading and running them on 1.2% (*w*/*v*) agarose gel electrophoresis at 80 V voltage for 2 h. They were visualized on the gel documentation system under UV light. The intact bands of 28S, 18S RNA on gel indicated that the isolated RNA was of a good quality. In addition, the absence of small RNA fragments clearly demonstrated that the integrity of the RNA was preserved. RNA samples from the leaves were present in lanes 1–6. All lanes showed good quality bands except lane 3 in which the concentration of RNA was reduced. In lane 4, a small adulteration of genomic DNA was observed (Figure 5).

### 2.5. qRT-PCR Data Interpretation

The relative changes in gene expression for the bioactive genes PSY and ZDS determined by real-time PCR are represented in Table 2. On the basis of the 2^−ΔΔCt^ method in both replicates of the leaves, the PSY and ZDS genes showed upregulation with two-fold changes. Quantitative PCR analysis exhibited that leaves had high amounts of zeta-carotene desaturase (ZDS) and phytoene synthase (PSY) beta carotenes as compared to the control. It showed that bioactive genes are more expressed with upregulation in the leaves of *Moringa oleifera* as compared to the control, and similar or higher than in *Brassica napus, Brassica rapa,* and *Brassica oleracea*, confirming the importance of *M. oleifera* plant leaves as medicinal plants.

## 3. Discussion

*M. oleifera* is a therapeutic plant with multiple antimicrobial characteristics. The medicinal activities of the extracts of *M. oleifera* are effective principally against several serious diseases. It was reported in several studies that *M. oleifera* is a good source of therapeutic proteins and bioactive components [17]. More recently, it has been revealed that the leaves of *M. oleifera* contain antioxidant agents which have the ability to alleviate degenerative conditions such as cardiovascular cancer, but their efficacy as antimicrobial agents against infectious diseases is relatively lower [18]. Similarly, another study reported that leaf extracts of *M. oleifera* have significant antioxidant and antimicrobial properties which confirm their potential as a cure against a wide variety of infectious diseases [19]

Compared to *Brassica napus*, *Brassica rapa, Brassica oleracea* and *Bixa orellana*, molecular studies of *M. oleifera* are not well reported. Computational analysis such as phylogenetic analysis, and multiple sequence analysis, showed that the bioactive beta carotene genes zeta-carotene desaturase (ZDS) and phytoene synthase (PSY) belong to similar ancestors in terms of evolutionary lineage. Meanwhile, multiple sequence alignment and Pfam database analyses revealed that the ZDS and PSY bioactive genes have common domains and protein families with *Arabidopsis thaliana, Brassica napus, Brassica rapa, Brassica oleracea* and *Bixa orellana*. In some studies, the association of a few antioxidant-linked enzymes in the vegetative parts of *M. oleifera*, such as catalase, Lascorbate peroxidase, peroxiredoxin and peroxidase, have been confirmed through both in vivo and in vitro analyses [20]. Computational analysis based on reference genes from *A. thalliana* showed the presence of ZDS and PSY genes in the *Brassica* family, which has several phytonutrients capable of inhibiting particular enzymes that usually stimulate carcinogens and that also nurture other enzymes which assist in the disassembly of active carcinogens [21]. Moreover, carotenoids are also essential compounds in human diets, primarily as a precursor of vitamin A. 

The molecular biology technique qRT-PCR quantified gene expression with the help of RNA and cDNA. The quantitative analysis showed that ZDS genes have upregulation in all the plant leaves, including *Brassica napus, Brassica rapa, Brassica oleracea, Bixa orellana* and *M. oleifera*. In both replicates of leaves, the ZDS and PSY genes showed high gene expression. qRT-PCR analysis revealed that the leaves have high potential of beta carotenes. Carotenoids are from a group of natural pigments resulting from the conventional isoprenoid biosynthetic pathway. The details of enzymes involved in reactions of the carotenoid pathway and other fundamental details have been well researched in *Arabidopsis thaliana* [22]. Similarly, genome sequencing of *B. rapa* has revealed that the leaves of numerous cultivars of *B. rapa*, which are yellow and orange, are abundant in prolycopene, lutein and β-carotene [23]. Various *B. rapa* varieties are reported to be rich in carotenoids, which are well known as good for human health [23].

Various molecular studies as well as transcriptomics studies have shown that ZDS and PSY genes play a role in facilitating the accumulation of carotenoids in large amounts in *B. napus* chromoplast-rich tissues [24]. *Brassica oleracea*, known as Chinese kale, is also a member of the *Brassicaceae* family and is widely found in Southeast Asia. Chinese kale is commonly cultivated for its bolting stems which are common edible parts and are tender, crispy and tasty [25]. Overall, the literature showed that ZDS and PSY are important genes in the biosynthesis pathways of carotenoids, not only in *A. thaliana*, but also in the abovementioned plants of the *Brassicas*. The quantitative gene expression analysis of ZDS and PSY and their high expression has not only corroborated their medicinal importance but also their role in carotenoid synthesis. Moreover, this study has determined that the *M. oleifera* plant has large amounts of ZDY and PSY, showing the importance of this common medicinal plant. 

Multiple studies have reported the anti-microbial and antioxidant activities of the leaves of *M. oleifera* because of the existence of terpenoids that are associated with the stimulation of β-cells and the consequent insulin secretion. Moreover, flavonoids have been revealed to play a significant part in hypoglycemic actions [26,27]. The magnitude of the problem was identifying the exact plant segment that has the bioactive genes, and which component of *M. oleifera* is the most significant in terms of its medical potential. Moreover, the dearth of knowledge about the *Moringa* segment containing the bioactive components limits the ability to estimate and examine its antibiotic, antiseptic and antioxidant effects.

## 4. Materials and Methods

### 4.1. Assembling the Genes Data Set

The assembled gene sequences of the bioactive compounds of *Arabidopsis thaliana*, zeta-carotene desaturase ZDS (Gene ID: 819647) and phytoene synthase PSY (Gene ID: 831587), were retrieved from National Center for Biotechnology Information (NCBI) using the NC_003074.8 and NC_003076.8 reference numbers, respectively. The BLAST analysis was conducted to find the similarity clustering with other plant species such as *Brassica napus, Brassica rapa,* and *Brassica oleracea* which are rich sources of ZDS and PSY. The highly similar gene sequences from these plants were retrieved for gene as well as protein analysis following functional characterization.

### 4.2. DNA Extraction, PCR and Sequencing of ZDS and PSY Genes

In order to verify and to look into the sequence variation compared to *A. thaliana*, the DNA from *M. oleifera* was extracted, amplified by PCR, and the gene amplicon was sequenced [28]. First of all, 0.2 g of *M. oleifera* fresh leaves was taken, crushed and ground using pestle and mortar, and added in a 1.5 mL Eppendorf tube containing 800 µL extraction buffer. The tube was incubated for 35–45 min at 65 °C, with inversion during incubation following the addition of chloroform: isoamyl alcohol (24:1, *v*/*v*) in equal volume, mixing 8–10 times, and centrifugation at 13,000 rpm for 15 min. The supernatant was taken and transferred to a new tube following the addition of absolute ice-cold isopropanol in equal volume and then centrifuged again at 13,000 rpm for 10 min. The supernatant was discarded and the pellet was washed with 70% (*v*/*v*) ethanol, air dried and dissolved in 100 µL TE buffer. PCR using ZDS and PSY gene primers was conducted, and PCR product was used for sequencing. The sequences of both genes’ amplicons were then submitted to NCBI for accession number.

### 4.3. Multiple Sequence Alignment and Phylogenetic Analysis

Multiple sequence alignments of ZDS and PSY gene sequences of bioactive beta carotenes were performed using Clustal W available in Molecular Evolutionary Genetic Analysis software (MEGA 7.0) [29]. Furthermore, the phylogenetic evolutionary relationship analysis of the beta carotene gene sequences of *M. oleifera*, *A. thaliana*, *Brassica napus*, *Brassica rapa* and *Brassica oleracea* was performed using MEGA 7. A phylogenetic tree was constructed using the maximum likelihood method provided in the list of phylogeny functions of MEGA7 software [29]. The evolutionary history of these sequences was represented by bootstrap consensus tree (from 1000 replicates), in a traditional branch style. Trees for the heuristic search were obtained by applying neighbor-join algorithms to a matrix of pairwise distances estimated using the maximum composite likelihood approach and then selecting the topology with superior log-likelihood value. The evolutionary distances were computed using maximum composite likelihood method and were in the units of the number of base differences per site. 

### 4.4. Protein Profiling and Analysis

The retrieved ZDS and PSY genes of *Brassica napus, Brassica rapa, Brassica oleracea* and *Arabidopsis thaliana* were searched against the Pfam 32.0 (https://pfam.xfam.org/ (accessed on 20 January 2022)) collection of hidden Markov model profiles for characteristics of a family [30]. Pfam contains 17,929 families, which include over 70% of the protein sequences of all plants. The collection of Pfam thresholds was selected using the lowest scores for gene sequences belonging to the family and the highest score for sequences not belonging to the family were used to give protein family classifications. It classified beta carotene gene sequences into families based on the sequence homologies related to similar functions and conserved structures. The domains of bioactive genes of all plants were figured out using the Conserved Domain Database (CDD) v 2.12 (https://www.ncbi.nlm.nih.gov/Structure/cdd/cdd.shtml (accessed on 10 February 2022)), which is a collection of well-annotated full-length domains. CDD v 2.12 contains 3078 NCBI-curated domains in 495 hierarchies, including 298 single-domain hierarchies and 197 trees with 2357 leaf and 423 internal domains. The structures of ZDS and PSY were predicted using I-Tasser, a server that is used for protein structure and prediction [31]. A cavity analysis was done in a predicted protein structure using the Protein, Plus tool.

### 4.5. Primer Design

Following the complete multiple sequence alignment as well as evolutionary analysis of ZDS and PSY for *Brassica napus, Brassica rapa, Brassica oleracea* and *Arabidopsis thaliana*, the PCR primers were designed using *A. thaliana* ZDS and PSY genes as the template (Table 3). The web-based program Primer 3 with its default settings was used for this purpose [32]. Primer 3web version 4.1.0 is commonly used to find multiple, high-scoring primers for each candidate gene [32]. The primers were designed in a manner to have melting temperatures within the ranges of 50 to 60 °C depending upon specific genes.

### 4.6. Sample Collection and RNA Extraction

The samples of leaves of *M. oleifera* were collected from a field (AlUla, Saudi Arabia), as reported earlier [33]. The fresh leaves were harvested and frozen in liquid nitrogen for total RNA extraction. The total RNA was extracted by Trizol method [34]. First of all, each sample of leaves was ground in the presence of liquid nitrogen. Samples were vortexed vigorously in the presence of stainless ball bearing beads to make powder. An amount of 1 mL TRIZOL reagent was added and kept on rotor mixer for 10 min and centrifuged up to 11,000× *g* for 10 min in cold room. After mixing thoroughly, the supernatants were transferred to new 1.5 mL tubes and 200 µL chloroform was added and they were shaken by hand for 15 s till incubation of both samples at room temperature for 3 min. They were centrifuged up to 11,000× *g* for 15 min in cold when RNA persisted in the colorless upper aqueous phase. The aqueous phase was transferred from each sample to a fresh tube and 200 µL isopropyl alcohol was added and centrifuged at 11,000× *g* for 10 min in cold till the RNA formed a gel-like pellet. The supernatant was discarded, and 1 mL of 75% ethanol was added, vortexed, centrifuged at 7500× *g* for 5 min at freezing temperature, and all ethanol was removed. Eppendorf tubes were kept open in air to dry the RNA pellet until the pellet turned from white to clear. The RNA was re-suspended in 25 µL RNase-free water by pipetting up and down, incubated for 10 min and the total RNA was stored at −70 °C.

### 4.7. cDNA Preparation

The cDNA was prepared using Thermo Scientific Revert Aid First Strand cDNA Synthesis Kit according to the protocol of the manufacturer’s manual [35]. Firstly, 1 µg of total RNA, 1 µL of 10× reaction buffer with MgCl_2_, 1 µL of DNase I and 10 µL of nuclease free water were added into an RNase-free tube and incubated at 37 °C for 30 min. An amount of 1 µL of 50 mM EDTA buffer was added and incubated at 65 °C for 10 min. The prepared RNA was used as template for reverse transcription. In a sterile nuclease-free tube, 2 µg of total RNA, 1 µL of Oligo(dT) primers (forward + reverse) and 12 µL of nuclease-free water were added and kept on ice. In a separate sterile tube, 4 µL of 5× reaction buffer, 1 µL of Ribo Lock RNase inhibitor (20 µL), 2 µL of 10 mM dNTP Mix and 1 µL of RevertAid RT (200 U/µL) were added and mixed gently using centrifuge. The mixture was incubated at 42 °C for 60 min and subjected to 700 rpm centrifuge for 5 min and stored at −70 °C.

### 4.8. qRT-PCR Analysis

The amplification of the cDNA in qRT-PCR was analyzed using a Thermo scientific Piko Real-Time PCR system (Thermo Fisher Scientific Inc., Waltham, MA, USA). The cDNA for each sample was added in the appropriate amount for the amplification reaction. The PCR analysis was conducted using Piko Real-Time PCR according to the manufacturer’s instructions [36]. Three samples of the leaves of *M. oleifera* were prepared as technical replicates having one reference gene or internal control of 10 pico mole/µL primers (primer sequences are given in Table 2). The SYBR green dye 12.5 µL and 1.6 µL primer mixture (forward + reverse) were mixed and added in each well of the PCR plate. In addition, 8.9 µL of nuclease-free water and 2 µL cDNA were added in each well of the PCR plate and mixed gently. The denaturation temperature of 95 °C was set for 15 s. The annealing and extension both were carried out at 60 °C, which is the optimal temperature for the amplification by Taq DNA polymerase but at the same time ensures more efficient cleavage of the probe. 

### 4.9. Quantitative Gene Expression Analysis

The fold change in gene expression was determined by the standard threshold cycle (Ct). The 2^−ΔΔCt^ method was used for relative quantification of bioactive genes in *M. oleifera*. Relative expression for all the target genes and the reference gene were calculated using the 2^−∆∆CT^ analysis method mentioned below [37]. It uses mathematical equations to calculate the relative expression level of the target gene as compared (relative) to the reference control and/or calibration. By using both calibrator and reference gene, the amount of target gene transcripts in a sample is first normalized with the reference genes and their expression is relativized to a normalized calibrator, according to the following formula [38]:

Relative Gene Expression =2^−ΔΔCq^

X-Test/X-Control= 2^−∆∆𝐶𝑇^ = 2(C_T, X_ − C_T, X_) _control_ − (C_T, X_ − C_T, R_)_test_

where ∆∆Cq = ∆Cq (sample) − ∆Cq (calibrator), ∆Cq = target gene Cq − reference gene Cq, while Cq = cycle quantification is usually known as Ct = cycle threshold. Likewise,

X test = Expression level of target gene. 

X control = Expression level of reference gene.

C_T, X_ = Target gene threshold cycles.

C_T, R_ = Reference gene threshold cycle.

It is very important to mention that biological and technical sample replications were carried out to conduct a statistical analysis, significantly evaluating gene expression and validating the results.

## 5. Conclusions

In the past few decades, substantial progress has been made regarding the acquaintance with bioactive compounds and their controlling genes in plants and their relations with human health. *Moringa oleifera*-based human medicines contain hundreds of bioactive components which are considered as nutrients and play a vital role in health maintenance. Without knowledge of the gene expression of the beta carotene bioactive compound, the treatment and therapeutics of several ailments seemed difficult to settle. The current research correlates with clinical genomics for *M. oleifera* by describing an interface of plant-based bioactive beta carotene genes, their expression and their curative benefits for human beings. This study highlighted the identification of zeta-carotene desaturase (ZDS) and phytoene synthase (PSY) genes in *Mongifera oleifera* using a computational genomics approach. Using the ZDS and PSY genes of *A. thaliana* as reference, BLAST analysis confirmed their presence in *M. oleifera* and other plants. These results were further validated by performing qRT-PCR analysis on RNA samples extracted from the leaves of *M. oleifera*. Nevertheless, these findings will need randomized clinical trials and population studies to suggest associations between the bioactive beta carotene genes of *M. oleifera* leaves and the cures for several diseases.

## Figures and Tables

**Table 1:** E-values, identities and domains found by using BLAST (Basic Local Alignment Search Tool), CDD (Conserved Domains Database) and Pfam (Protein families).

Species Name	Gene Name	E-Value	Identity (%)	Domain	Family
*Arabidopsis thaliana*	ZDS	0.0	98.0	PLN02487	Amino oxidase
	PSY	0.0	97.5	PLN02632	Squalene Synthase (SQS)
*Brassica napus*	ZDS	1 × 10^−121^	83.4	PLN02487	Amino oxidase
	PSY	1 × 10^−140^	83.5	PLN02632	Squalene Synthase (SQS)
*Brassica rapa*	ZDS	1 × 10^−11^	76.4	PLN02487	Amino oxidase
	PSY	1 × 10^−148^	82.3	PLN02632	Squalene Synthase (SQS)
*Brassica oleracea*	ZDS	1 × 10^−13^	86.0	PLN02487	Amino oxidase
	PSY	1 × 10^−148^	83.5	PLN02632	Squalene Synthase (SQS)

**Table 2:** ZDS and PSY gene expression using quantitative real-time PCR in *Brassica napus, Brassica rapa, Brassica oleracea* and *Bixa orellana*.

Genes	Expression Changes Relative to That of *A. thaliana*				
	*Arabidopsis thaliana*	*Brassica napus*	*Brassica rapa*	*Bixa orellana*	*Moringa oleifera*
ZDS	↑651.3 *	↑412.8 *	↑615.0 *	↓140.8 *	↓140.8 *
PSY	↓412.3 *	↑536.1 *	↓341.8	↓514.1 *	↓514.1 *
Actin	↑120.9 *	↑102.6 *	↑7.4 *	↓27.0 *	↓17.0 *

↑: upregulation; ↓: downregulation; *: the change in gene expression is more than two-fold compared to the universal control actin.

**Table 3:** List of primers of bioactive genes designed by Primer 3.0 tool.

S. No.	Genes	Primers	Length	T_M_ (°C)	GC (%)
1.	PSY	Forward. TGCAGCTTAAACGAGCAAGA	20	59.90	45
		Reverse. AGCAATGAAGCCCATACCTG	20	60.10	50
2.	ZDS	Forward. GACTCCGATGTTTCCGACAT	20	59.93	50
		Reverse. CACTTTGCCACCAATGAATG	20	59.96	45

## Data Availability

All the data are presented in this manuscript.

## Sample Availability

Samples of the DNA and primers are available from the authors.
